# M2 receptors activation modulates cell growth, migration and differentiation of rat Schwann-like adipose-derived stem cells

**DOI:** 10.1038/s41420-019-0174-6

**Published:** 2019-05-03

**Authors:** Roberta Piovesana, Alessandro Faroni, Valerio Magnaghi, Adam J. Reid, Ada Maria Tata

**Affiliations:** 1 Department of Biology and Biotechnologies “Charles Darwin”, “Sapienza” University of Rome, Rome, 00185 Italy; 2 Blond McIndoe Laboratories, Division of Cell Matrix Biology and Regenerative Medicine, School of Biological Sciences, Faculty of Biology, Medicine and Health, The University of Manchester, Manchester Academic Health Science Centre, Manchester, M13 9PT UK; 3 Department of Pharmacological and Biomolecular Sciences, University of Milan, Milan, 20133 Italy; 4 Department of Plastic Surgery & Burns, Wythenshawe Hospital, Manchester University NHS Foundation Trust, Manchester Academic Health Science Centre, Manchester, UK; 5 Research Center of Neurobiology “Daniel Bovet”, “Sapienza” University of Rome, Rome, 00185 Italy

**Keywords:** Peripheral nervous system, Stem-cell research

## Abstract

Schwann cells (SCs) play a central role in peripheral nervous system physiology and in the response to axon injury. The ability of SCs to proliferate, secrete growth factors, modulate immune response, migrate and re-myelinate regenerating axons has been largely documented. However, there are several restrictions hindering their clinical application, such as the difficulty in collection and a slow in vitro expansion. Adipose-derived stem cells (ASCs) present good properties for peripheral nerve regenerative medicine. When exposed to specific growth factors in vitro, they can acquire a SC-like phenotype (dASCs) expressing key SCs markers and assuming spindle-shaped morphology. Nevertheless, the differentiated phenotype is unstable and several strategies, including pharmacological stimulation, are being studied to improve differentiation outcomes. Cholinergic receptors are potential pharmacological targets expressed in glial cells. Our previous work demonstrated that muscarinic cholinergic receptors, in particular M2 subtype, are present in SCs and are able to modulate several physiological processes. In the present work, muscarinic receptors expression was characterised and the effects mediated by M2 muscarinic receptor were evaluated in rat dASCs. M2 receptor activation, by the preferred agonist arecaidine propargyl ester (APE), caused a reversible arrest of dASCs cell growth, supported by the downregulation of proteins involved in the maintenance of cell proliferation and upregulation of proteins involved in the differentiation (i.e., c-Jun and Egr-2), without affecting cell survival. Moreover, M2 receptor activation in dASCs enhances a pronounced spindle-shaped morphology, supported by Egr2 upregulation, and inhibits cell migration. Our data clearly demonstrate that rat dASCs express functional muscarinic receptors, in particular M2 subtype, which is able to modulate their physiological and morphological processes, as well as SCs differentiation. These novel findings could open new opportunities for the development of combined cell and pharmacological therapies for peripheral nerve regeneration, harnessing the potential of dASCs and M2 receptors.

## Introduction

Peripheral nerve injuries (PNIs) derived from mechanical trauma or surgical resection secondary to tumour excision are frequent in human. Although the peripheral nervous system (PNS) has a good regeneration capability, several factors can influence nerve repair such as the type and location of injury. However, nerve regeneration outcomes are rarely satisfactory and full functional recovery is often limited^1^. Following injury, neurons and Schwann cells (SCs) change their cellular and molecular properties in order to create a pro-regenerative environment^2^. Several studies reported that SCs played a relevant role in peripheral regeneration. Regeneration of injured nerves depends on SCs response to produce growth factors, which convert their phenotype into a c-Jun-mediated phenotype called *repair SCs*^3^. SCs support nerve regeneration by downregulating myelin genes, upregulating trophic factor production, and increasing cytokine release, all resulting in myelin clearance through a process called myelinophagy, which is mediated by both SCs and macrophages^4^. Finally, SCs form the Büngner’s bands which drive regenerating axons towards their targets^3^.

Although SCs role is pivotal after nerve injury, they are not sufficient to support a complete and efficient recovery of peripheral functions clinically. Adipose-derived stem cells (ASCs) may represent an interesting tool for regenerative medicine^5,6^, considering their ability to differentiate into Schwann-like cells (differentiated adipose-derived stem cells, dASCs) under appropriate conditions^7,8^. After 18 days of differentiation with selective growth factors, dASCs show properties similar to SCs and they have the ability to improve neurite outgrowth both in vitro and in rodent in vivo models^1^.

Glial cells express receptors for neurotransmitters (i.e., GABA, ATP, acetylcholine), which can modulate cell growth and differentiation^9–13^. Acetylcholine (ACh) involvement in the cross-talk between neurons and glia during neurogenesis, as well as in the adult nervous system, has been widely reported^14,15^.

Previous studies demonstrated that rat SCs express cholinergic muscarinic receptors. In particular, the most abundant receptor subtype is M2^9,16^.

Muscarinic acetylcholine receptors are G-protein coupled receptors and, in particular, M2 and M4 receptors are coupled with Gi protein, which causes the inhibition of cyclic AMP (cAMP) production and K^+^ channels modulation^14^.

In SCs, the selective activation of M2 receptors causes a reversible inhibition of cell proliferation, differentiating SCs towards a promyelinating phenotype expressing myelinating transcriptional factors such as Sox10 and Krox20. At the same time the expression of proteins involved in the maintenance of the undifferentiated state (e.g., c-Jun, Notch-1, and Jagged-1)^16^ is suppressed. Myelin alterations and axon degeneration have been also observed in sciatic nerves of M2/M4 KO mice^10^.

dASCs, like SCs, express functional receptors for different neurotransmitters^17–19^. Previously, it has been demonstrated that adipose mesenchymal stem cells (ASCs) are cholinoceptive, expressing different muscarinic receptor subtypes, and in particular, M2 subtype^20^. Recent study shows dASCs express choline acetyltransferase, vesicular acetylcholine transporter and secreting ACh; they promote proliferation of myoblast^21^. In the present work, we characterised the expression of muscarinic receptors in dASCs and in particular the effects mediated by M2 receptor activation in terms of cell proliferation and migration. In dASCs, similarly to what observed in SCs, M2 receptor activation caused a reversible decrease of cell proliferation and the inhibition of cell migration. Furthermore, the stimulation of M2 receptors enhanced a pronounced spindle-shaped morphology acquired after growth factor induction, resembling native SCs morphology. Therefore, M2 muscarinic receptor could be a new pharmacological target to regulate important physio-pathological events aimed at improving the recovery from of peripheral nerve injuries.

## Results

### Expression of stem cell markers and muscarinic receptors

**Fig. 1:**
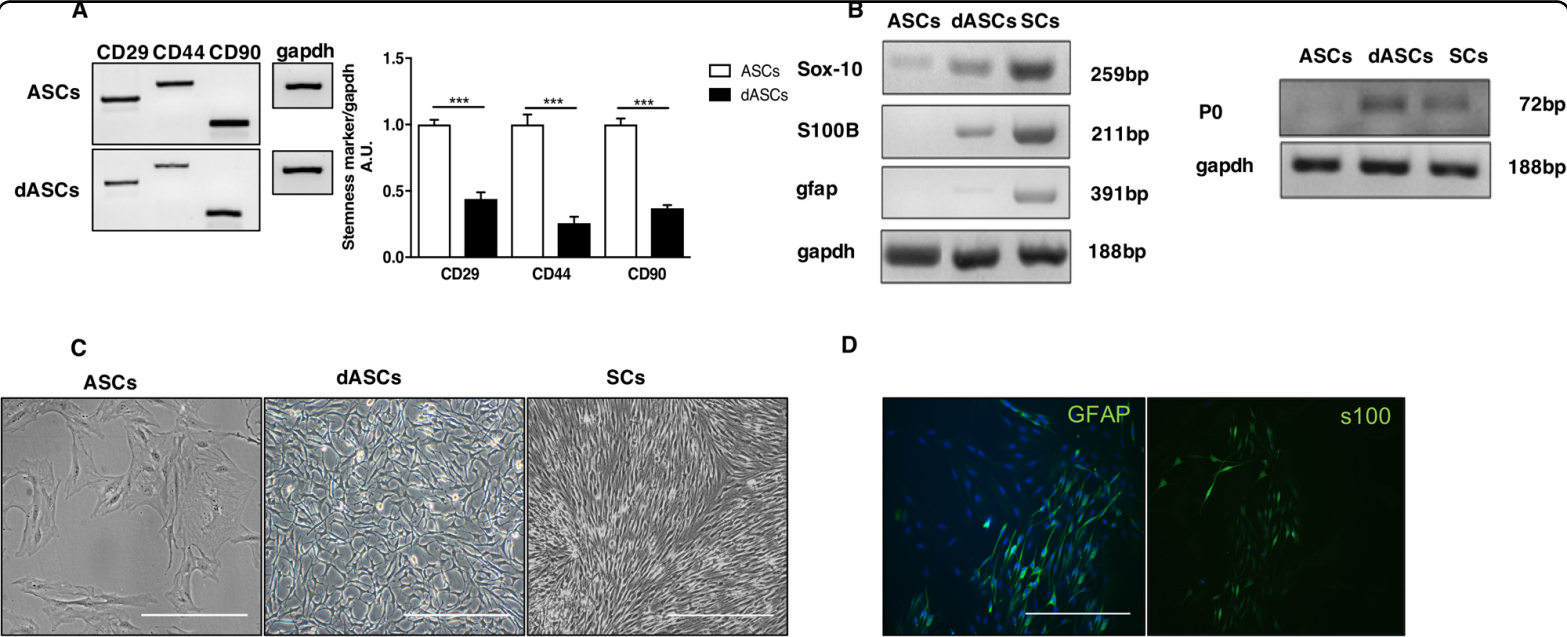
Stem cell and glial markers expression. **a** Stem cell markers expression was assessed by RT-PCR. ASCs were used as positive control for stem cell markers expression. After growth factors exposure, stemness markers, CD90, CD44 and CD29 were significantly downregulated in dASCs (***p < 0.001). Data are represented as mean ± SEM of three independent experiments. **b** Glial markers expression was evaluated by RT-PCR. ASCs were used as negative control, while nerve-derived SCs were used as positive control. After 18 days of differentiation, glial markers were upregulated in dASCs (i.e., Sox10, S100β, GFAP and P0). **c** Analysis in contrast phase microscopy showed that dASCs changed their morphology compared to ASCs, featuring the classical spindle-shaped morphology similar to SCs (scale bar: 200 µm). **d** Expression of S100β and GFAP in dASCs was analysed by immunocytochemistry (scale bar: 100 µm)

ASCs express several stem cell markers such as CD29, CD44, CD54 and CD90^20,25,26^. Firstly, to confirm dASCs differentiation, CD29, CD44 and CD90 expression was evaluated by RT-PCR analysis both in ASCs and dASCs. As expected, after selective growth factors exposure, dASCs showed a significantly downregulated expression of stem cell markers (***p < 0.001; Fig. 1a). At the same time dASCs significantly increased the expression of some glial markers such as S100β, Sox10, GFAP and P0 (Fig. 1b). Moreover, after differentiation, they assumed elongated spindle-shaped morphology, similar to SCs (Fig. 1c) and, as demonstrated by immunostaining, 70% of the cells express SCs proteins such as GFAP and S100β (Fig. 1d).

**Fig. 2:**
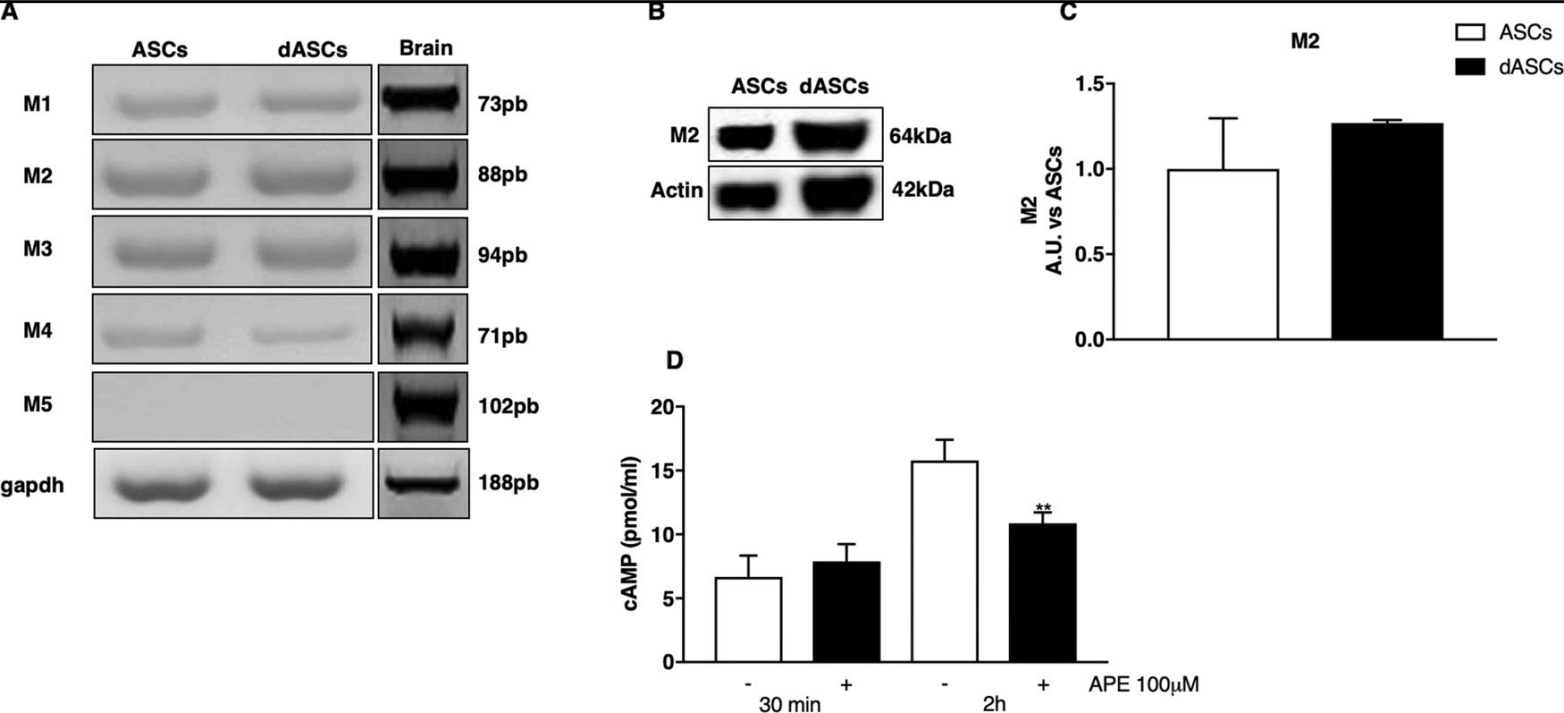
Muscarinic receptor subtypes expression. **a** dASCs, as well as undifferentiated ASCs express all muscarinic receptors transcripts, except M5. GAPDH was used as housekeeping gene and brain lysates were used as positive controls. **b** Western blot showed M2 muscarinic receptor expression in ASCs and dASCs. β-actin was used as protein reference. **c** Densitometric analysis did not show any particular variation of M2 protein levels after SCs phenotype induction. Data are represented as mean ± SEM of three independent experiments and normalised against ASCs as control group. **d** Levels of cyclic AMP (cAMP) in dASCs. cAMP levels decreased after 2 h of treatment with APE 100 µM compared to untreated controls (**p < 0.01)

Muscarinic receptor expression was characterised in dASCs by RT-PCR analysis, demonstrating that they express the transcripts for m1, m2, m3 and m4 subtypes (Fig. 2a). Giving the importance of M2 muscarinic receptor activation in SCs^9,10,16^, M2 muscarinic receptors expression was also investigated by western blot analysis, indicating this receptor subtype is already expressed in ASCs, as previously demonstrated^20^, and its expression is not significantly different in dASCs (Fig. 2b, c).

We also demonstrated that APE 100 µM (Arecaidine Propargyl Ester) treatment, activating selectively M2 receptor, induced a significant decrease of cAMP levels in the cells, showing that M2 receptor in dASCs is functional (Fig. 2d). Indeed, APE is able to significantly decrease cAMP levels after 2 h of treatment, but not following only 30 min of exposure.

### M2 receptor selective activation decreased cell growth

To investigate the downstream effects mediated by M2 muscarinic receptor activation, cells were treated with APE up to 7 days. APE was used at the final concentration of 100 µM, the same concentration used previously in SCs and in ASCs experiments^10,20^.

**Fig. 3:**
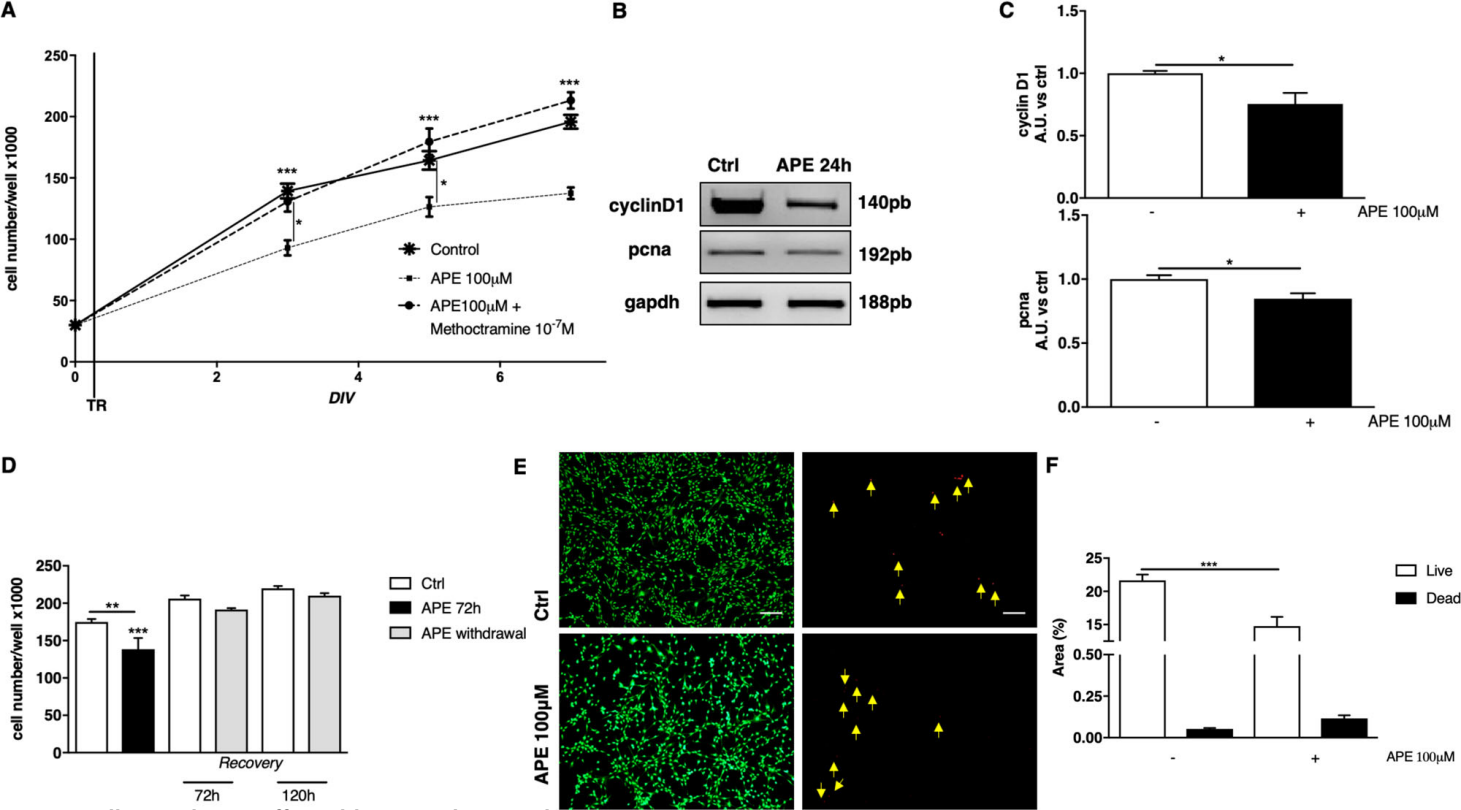
Cell growth was affected by M2 subtype selective activation. **a** Cell growth was analysed by MTS assay. M2 agonist treatment, APE 100 μM, was maintained from 3 to 7 days. M2 activation caused a decreased of cell growth (***p < 0.001). Cell growth was also evaluated in cultures maintained in the presence of APE 100 μM plus M2 antagonist (10^−7^ M Methoctramine; Meth), which was able to counteract APE effect. **b**, **c** The decreased cell growth was accompanied by a downregulated expression of cyclin D1 and PCNA transcripts (24 h of APE 100 μM treatment)(*p < 0.05). **d** Recovery of proliferative capability was assessed after 72 h of APE treatment (100 μM). APE was removed and the cell growth was analysed until 120 h by MTS assay. dASCs were able to recover cell proliferation after APE withdrawal. The results are the mean ± SEM of three independent experiments performed in triplicate (**p < 0.01 and ***p < 0.001). **e**, **f** The analysis of cell viability was performed after 48 h of APE treatment. There was no significant increase in the percentage of dead cells after APE treatment. Images were taken at 4x magnification (scale bar: 200 µm) and the dead cells are highlighted with yellow arrows. (***p < 0.001). Data are represented as mean ± SEM of three independent experiments

After APE treatment, dASCs cell growth decreased significantly over 7 days in vitro (*DIV*, Fig. 3a). Moreover, to further confirm that the cell growth inhibition was specifically mediated by M2 receptor, the M2 antagonist methoctramine was used. The results indicated that 10^−7^ M methoctramine was able to counteract APE effect (Fig. 3a). Indeed, in the presence of the M2 antagonist, APE was not able to decrease dASCs growth and the number of the cells remained comparable to the untreated cells. The decrease in cell growth was also confirmed by the significant downregulation of genes involved in the control of the cell cycle (i.e., pcna and cyclin D1) (Fig. 3b, c).

The decreased cell growth was reversible. Indeed, after APE withdrawal, dASCs were able to rescue cell proliferation. In absence of M2 agonist, dASCs were able to recover their ability to proliferate, reaching levels of cell number comparable to untreated controls (Fig. 3d).

In order to exclude the possible toxic effect of APE in dASCs, Live-Dead Assay was performed. The analysis of cell viability confirmed that the total cell number is decreased following APE treatment, as shown by the significant reduction of live cells stain (green, Fig. 3e, f). However, the percentage of dead cells (red) was not significantly modified by the M2 agonist treatment, demonstrating that APE 100 μM treatment is not cytotoxic for dASCs (Fig. 3e, f).

### M2 receptor activation increased the spindle-shaped morphology and SC-marker expression

**Fig. 4:**
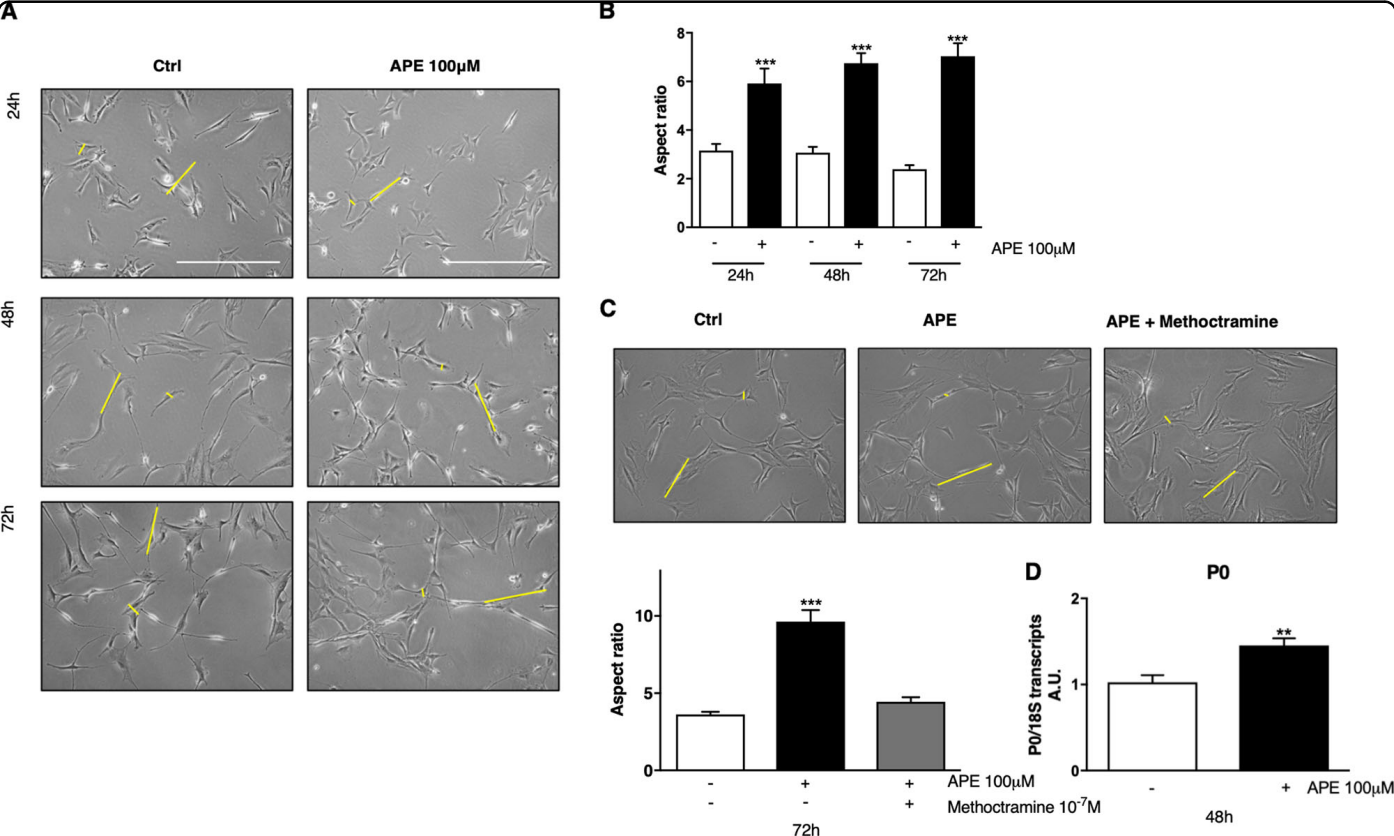
Morphological changes following M2 receptor selective activation. **a** Following APE treatment (24, 48 and 72 h of treatment) dASCs appeared at lower density compared to untreated cells and presented a more elongated, spindle-shaped morphology (scale bar: 200 µm). Representation of the measured lengths is highlighted by the yellow lines. **b** dASCs treated with APE 100 µM exhibit more spindle-like shape with an increase of aspect ratio when compared to untreated cells (***p < 0.001). **c** To confirm that modified cell morphology was the result of M2 selective activation, the same experiment was performed in presence of 10^−7^ M of M2 antagonist Methoctramine plus APE 100 µM. M2 antagonist was able to counteract APE effect. Aspect ratio in presence of antagonist was comparable to the untreated cells (***p < 0.001). Data are represented as mean ± SEM of three independent experiments. **d** APE treatment upregulated P0 transcripts expression (**p < 0.01)

After differentiation, dASCs present a typical spindle-shaped morphology^1,7,27^. APE treatment caused a more evident elongated morphology already after 24 h of treatment (Fig. 4a, b). After APE treatment, the mean cell diameter of the dASCs decreased and the length increased. The results were graphed as *Aspect Ratio*, the ratio between the cell length and diameter (Fig. 4b). This ratio significantly increased after 24 h of APE treatment and progressively increased with the time of treatment. To confirm that aspect ratio increase was dependent on M2 receptor activation, the same experiment was also performed after 72 h of APE treatment and in presence of M2 antagonist methoctramine (10^−7^ M). When M2 antagonist was present, dASCs morphology was similar to the untreated cells and the aspect ratio remained comparable to that of the untreated cells (Fig. 4c). Furthermore, a significant upregulation of myelin protein P0 transcript, a classic SCs marker, corroborates these findings, suggesting improved SCs differentiation (Fig. 4d).

### M2 receptor stimulation decreased proliferative related genes and increased egr-2 protein

**Fig. 5:**
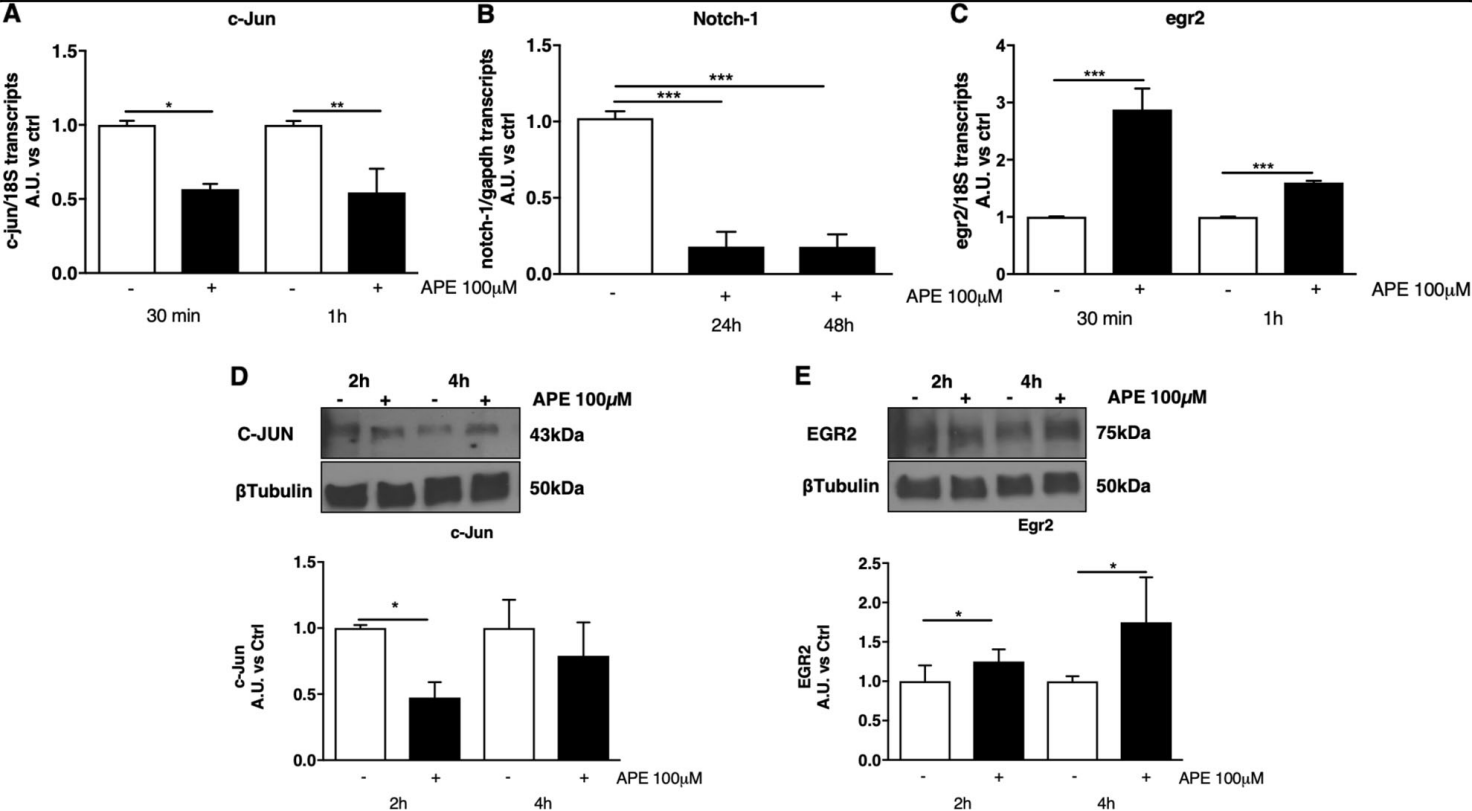
M2 receptor activation was able to regulate dASCs proliferation and differentiation markers. **a**, **b** Analysis by qRT-PCR demonstrated that APE 100 µM negatively downregulated c-jun and notch-1 transcripts (*p < 0.05, **p < 0.01 and ***p < 0.001). **c** APE 100 µM induced an upregulated expression of egr-2 mRNA at both 30 minutes and 1 h (***p < 0.001). **d**, **e** The transcriptional factors c-Jun and Egr-2 were also evaluated by western blot analysis. β-tubulin was used as protein reference. The expression of Egr-2 appears significantly upregulated after 4 h of treatment while c-Jun was significantly downregulated after 2 h of APE treatment (*p < 0.05). Data are represented as mean ± SEM of three independent experiments

In order to validate that the decreased cell number was associated with decreased cell proliferation and possible differentiation, qRT-PCR was used to assess the gene expression changes of some proliferation and differentiation markers. As indicated in Fig. 5a, b, APE treatment was able to significantly decrease c-jun and notch-1 expression. Interestingly, the decreased expression of c-jun and notch-1 was accompanied by an increased expression of egr-2, a gene associated to SCs differentiation (Fig. 5c). These findings were also confirmed at the protein level. Indeed, M2 selective activation was able to reduce the levels of c-Jun protein significantly after 4 h and to upregulate Egr-2 protein after 2 h of treatment (Fig. 5d, e).

**Fig. 6:**
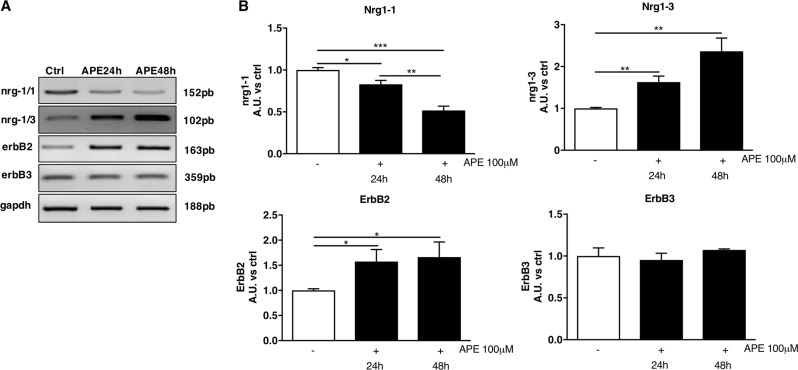
Neuregulins (NRG) and their receptors were regulated by M2 muscarinic receptors. **a** Analysis by RT-PCR indicated that APE 100 µM was able to downregulate NRG1-1 and upregulate NRG1-3 isoform transcripts. Moreover, M2 agonist treatment caused also an increase of erbB2 NRG receptor expression while any particular variation of erbB3 expression was observed. **b** The graphs show the densitometric analysis of the band normalised for the housekeeping gene GAPDH. The results are the mean ± SEM obtained from three independent experiments (*p < 0.05, **p < 0.01 and ***p < 0.001)

Neuregulins (NRG) are proteins expressed by axons and involved in neuron-glia cross talk and some authors described SCs ability to produce NRG-1 in absence of axons^28^. In particular, several isoforms are produced by alternative splicing: NRG1-1 is expressed in a proliferative state, while NRG1-3 isoforms are expressed in myelinating state. By using RT-PCR, we demonstrated that dASCs express NRG1-1 and NRG1-3 isoform transcripts (Fig. 6a). In accordance with MTS results, APE treatment caused a decrease of NRG1-1 transcript expression and an upregulation of NRG1-3, suggesting a switch between a proliferative and a differentiated state (Fig. 6a, b). Moreover, M2 agonist treatment caused an increase of NRG receptor expression, in particular the erbB2 subtype. Conversely, erbB3 receptors transcript levels were not modified by APE treatment (Fig. 6a, b).

### M2 receptors selective stimulation altered cell migration

**Fig. 7:**
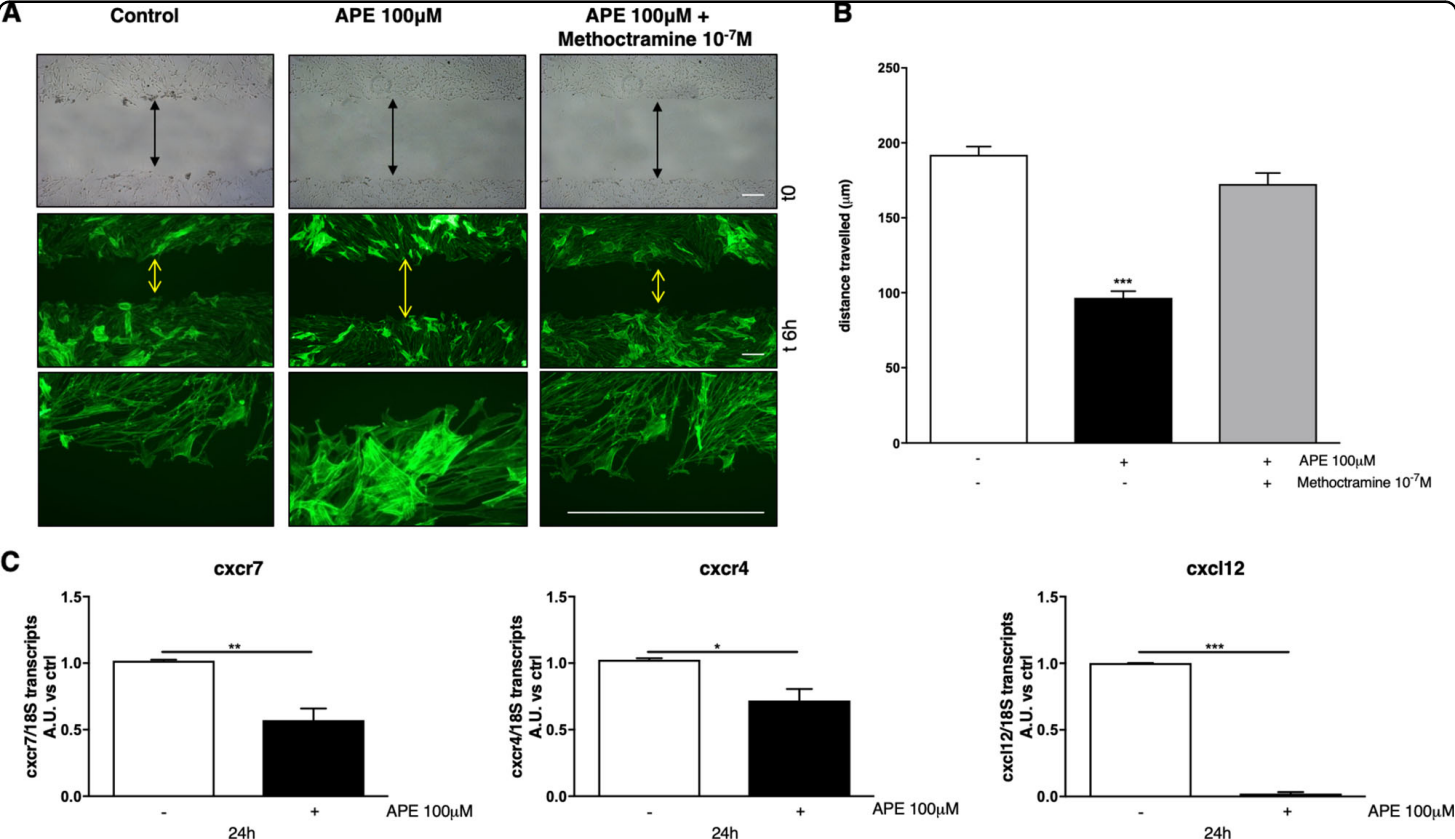
dASCs migration was reduced following M2 receptor stimulation. **a** Wound healing experiments were set up to study cell migration. The images were obtained at time 0 and after 6 h from scratch. Upper panel shows the images at phase contrast, whereas lower panels show the cells stained with fluorescent actin-specific dye (Phalloidin) at two different levels of magnification (scale bar: 200 µM). The distance of the gap between two fronts was measured at the two-time points. M2 activation was able to reduce dASCs migration (***p < 0.001). This event was likely due to M2 selective activation since following antagonization of this receptor with 10^−7^ M Methoctramine, cell migration was not modified, and migration was similar to untreated cells. Phalloidin staining showed that more stress fibres were present after APE treatment. **b** Graph showing quantitative measurements of the distance travelled (µm) (t0−t6 h). **c** Analysis by qRT-PCR of CXCL4/7-CXCL12 transcripts. APE (100 μM) treatment induced a downregulation of CXCL12-CXCR4/CXCR7 mRNA. These results are the mean ± SEM of three independent experiments (*p < 0.05; **p < 0.01; ***p < 0.001)

dASCs migration was analysed after 6 h by wound healing assay. The results indicated that the presence of M2 agonist APE negatively modulated cell migration. Migration was also measured in presence of methoctramine; M2 antagonist was able to counteract the APE effect and migration was comparable to untreated cells (Fig. 7a, b). After APE treatment, more stress fibres (shown by green Phalloidin staining, Fig. 7a) were present in treated dASCs compared to controls, supporting the decreased migration.

The CXCL12-CXCR7/4 pathway is usually implicated in the control of cell migration and proliferation. In order to explain the M2-mediated mechanisms responsible of cell migration arrest, we analysed the mRNA levels of SDF1/CXCL12 and its receptors CXCR4/CXCR7 by qRT-PCR analysis. The results demonstrated that M2 agonist treatment caused a significant downregulation of the expression of both chemokine transcripts and its receptors after 24 h of treatment (Fig. 7c).

## Discussion

dASCs derived from ASCs differentiation represent a promising intervention to support and guide peripheral nerve regeneration.

ACh controls neurite outgrowth and, via M2 muscarinic receptors, SCs development^10,16^.

The data reported in the present work clearly demonstrate that dASCs are cholinoceptive and that M2 receptor activation in dASCs produced similar effects to that observed in SCs. The reversible inhibition of cell proliferation, the improved spindle-shaped morphology and the inhibition of cell migration, together with the upregulated expression of SCs differentiation markers such as Egr-2, suggest that the M2 receptor stimulation may strengthen the dASCs spindle-like phenotype and promote differentiation to a SC-like phenotype.

Although the PNS has regenerative capacity, conventional treatments including surgical repair of the damaged nerves, do not achieve complete functional recovery.

The development of novel strategies to enhance peripheral nerve regeneration is, therefore, of great relevance^1^.

Peripheral nerve regeneration is dependent on the greater permissive environment which is likely provided in part by SCs. These cells have a repertoire of physiological roles, including protection and regulation of nutritive exchanges with axons, myelin production and digestion of axons and myelin debris, under pathological conditions. Moreover, SCs promote peripheral nerve regeneration due to their ability to proliferate, migrate and release growth factors driving axon elongation^29,30^. Despite the relevant roles played by SCs during peripheral nerve regeneration, their use in clinical applications is rather limited. Although dASCs may represent an excellent alternative to SCs, they quickly revert their phenotype when chemical stimulation is withdrawn^27^.

Considering the effects mediated by M2 muscarinic receptor activation in SCs^10,16^ and in ASCs^20^, it seems a relevant tool to characterise M2 receptors activation also in dASCs. These cells combined with novel treatments may represent a successful strategy in the clinical treatment of peripheral nerve lesions^31^.

Unlike other neurotransmitter receptors, in this manuscript, we showed that M2 receptor expression did not change following dASCs differentiation compared to ASCs. It is known that M2 receptor is able to reduce cAMP concentration via Gi-protein^32^. Our previous studies have clearly demonstrated by pharmacological experiments and M2 knockdown that APE selectively binds only M2 receptors^10,33^. APE treatment was able to reduce cAMP concentration, supporting the functional presence of M2 receptors on dASCs membrane.

Similar to observations in SCs, the pharmacological activation of M2 muscarinic receptors reduced cell growth, without influencing cell viability. This was demonstrated by proliferation and live-dead studies, and corroborated by the downregulation of genes involved in the control of cell cycle (i.e., pcna and cyclinD1).

The inhibitory effect of M2 agonist on cell proliferation was however reversible; APE withdrawal from culture medium caused a rescue of dASCs proliferation, confirming that M2 activation maintained cell plasticity, as observed in SCs and ASCs^16,20^.

SCs morphology depends on the physiological state of the cells; they change their morphology in order to participate at different physiological and pathological events^34^. The chemical differentiation via growth factors changes ASCs morphology, decreasing the cell diameter and improving cell length, in a phenotype that resembles native SCs.

Interestingly, M2 receptor activation caused a significant change in dASCs cell morphology, stimulating a more evident spindle-like phenotype. It is possible to speculate that these changes are a sign of occuring differentiation events. In this manuscript, we showed that dASCs expressed NRG1 isoforms transcript. NRG1 is an axonal factor, that supports nerve regeneration^35^; SCs are also able to produce this factor in autocrine way supporting survival and proliferation also in the absence of axons^28^. Indeed, several studies showed that SCs recapitulate a nerve injury condition in vitro^36^. Moreover, NRG1 is able to control many aspects of SCs differentiation and myelination^37^. It has recently been shown that withdrawal of NRG1-1 in dASCs decreased the expression of SCs markers^38^, and it is able to improve SCs-like phenotype in association with other growth factors (e.g., FGF, PDGF and Fsk)^39^. We showed that M2 stimulation upregulated NRG1-3, the isoform involved in the myelinating phenotype, suggesting an improvement in the SC-like phenotype. Our studies clearly demonstrated an upregulation of NRG, followed by an early upregulation of Egr2/Krox20 expression. We have previously shown that NRG alone can increase Egr2/Krox20 expression without M2 stimulation^39^, nevertheless, in this study we show that the synergistic action of NRG and M2 agonist further increases Egr2/Krox20 expression.

There is much evidence to indicate that SCs differentiation in myelinating phenotype depends on Krox-20/Egr2 and other promyelin transcription factors, that are normally activated by axonal signals^40^. Moreover, c-Jun is an important regulator of SCs plasticity and it is able to inhibit myelin related genes^3^. It is interesting that c-Jun and Egr-2 show a cross-antagonistic functional relationship. In our studies, we demonstrated that M2 selective activation was able to downregulate c-Jun after 2 h of treatment. Interestingly, this downregulation was accompanied by an upregulation of Egr-2, as described in the literature. This result supports the hypothesis that these cells respond in a similar way to SCs under physiological conditions; moreover, it is shown that ACh, via M2 receptor, is able to regulate their physiology, as indicated by morphological changes related to Egr-2 upregulation.

Another important event in the injury response is SCs migration. SCs are able to proliferate and then migrate to the injured site to help environmental clearance and nerve regeneration^41^. Other authors described that ACh stimulation in the presence of calcium enhanced migratory capacity via M1 subtypes in mesenchymal stem cells^42^. Herein, we showed that APE treatment, via M2 receptor stimulation, caused a decrease of cell migration in dASCs. In particular, after APE treatment, cells reorganised their cytoskeleton, showed a decreased cell migration, accompanied by a downregulated expression of CXCL12/SDF-1 and its receptors CXCR7 and CXCR4. This pathway is involved in the control of SCs migration during regeneration^43^ and dASCs also express and secrete chemokines and their receptors that modulate their migration activity^44^. In accordance with the observed inhibitory effect of APE on dASCs migration, the expression levels of CXCL12/SDF-1 and its receptors CXCR7 and CXCR4 were downregulated.

Albeit the ability of APE to selectively activate M2 receptor subtype has been largely demonstrated in our previous works^15,16,20,33^, to confirm APE specificity for M2 receptor, we performed co-treatments with the specific M2 antagonist methoctramine. The results obtained for the analyses of cell growth and cell migration, as well as the study of dASCs morphology, demonstrated that the presence of M2 antagonist counteracted the APE effects.

Altogether, these findings agree with reports in other papers and raise the possibility of other neurotransmitter receptors (i.e. GABA and purinergic receptors) to modulate ASCs and dASCs physiological processes^18,19,39^. In this scenario, neurotransmitter receptors could be potential pharmacological targets to modulate ASCs physiology and differentiation towards dASCs phenotype.

Although further analyses are needed to fully understand the role of M2 receptor in differentiation and the role of the other muscarinic receptor subtypes in dASCs, our data suggest that the activation of muscarinic receptors influences the physiology of the dASCs and could potentially represent a therapeutic target for nerve regeneration. In this context, we believe that these findings could lead to a novel strategy for nerve repair, combining the potential of stem cell therapy together with the pharmacological stimulation of neurotransmitters receptors on transplanted ASCs.

## Materials

### Adipose-derived stem cells harvesting and culture

The experimental procedures that involved animals were performed in accordance with the Animals (scientific procedures) Act 1986. ASCs were isolated from adult Sprague-Dawley rats as described previously^7^. Fat pads were dissected and chopped using a sterile razor blade. After, tissue was enzymatically digested for 1 h at 37 °C using 0.15% (w/v) collagenase type I (Invitrogen, UK). A 100 µm filter was used to remove the undissociated tissue. The solution was centrifugated at 1200 rpm for 10 min, and the stromal vascular fraction (SVF) was obtained. The resultant SVF includes preadipocytes, endothelial cells, macrophages, fibroblasts and adipose-derived stem cells (ASCs), which are able to adhere to plastic and proliferate rapidly than the other cell type^22^. The stromal cell pellet was plated in 75 cm^2^ cell culture flasks in stem cell growth medium consisting in alpha minimum essential medium (α-MEM) supplemented with 10% *(v/v)* fetal bovine serum (FBS), 2mM L-glutamine and 1% (v/v) Penicillin/Streptomycin solution. Cultures were maintained at subconfluent levels in a 37 °C incubator with 5% CO_2_.

### Stem cells differentiation to Schwann-like phenotype

For differentiation to SCs phenotype, at passage 1–2 ASCs were treated with stem cell growth medium supplemented with 1 mM β-mercaptoethanol for 24 h. The next day, cells were incubated with 10 ml of preconditioning medium for 72 h containing 35 ng/ml all-trans-retinoic acid at 37 °C. Following all-trans-retinoic acid treatment, cells were washed carefully and stem cell medium was replaced supplemented with 14 µM forskolin, 192 ng/ml glial growth factor-2 (GGF-2, Acorda, UK), 5 ng/mL platelet-derived growth factor (PDGF, Peprotech, USA), and 10 ng/ml basic fibroblast growth factor (bFGF Peprotech, USA) for 14 days^23^. The same supplemented medium was used for cell maintenance. Cells were incubated at humidified 37 °C environment with 5% CO_2_. SCs cultures were obtained from sciatic nerves of P1-P2 Sprague-Dawley rats using a previously established protocol^17,24^ and used as positive controls for SC-like differentiation.

### Experimental set up and pharmacological treatments

M2 muscarinic receptor agonist, Arecaidine propargyl ester hydrobromide (APE, Sigma-Aldrich, St. Louis, MO, USA) was used at the final concentration of 100 µM, according to previous studies^10,20^. M2 muscarinic receptor antagonist, methoctramine, was used at final concentration of 10^−7^ M (Meth, Sigma-Aldrich, St. Louis, MO, USA). M2 muscarinic receptor antagonist was added 2 h before APE treatment. Controls were obtained maintaining the cells in normal growth medium. Technical and experimental triplicates were performed for all experiments.

### RT-PCR and quantitative real-time PCR (qPCR)

Cells were collected at the time point chosen and stored in RNA cell protect agent (Qiagen, Manchester, UK). Total RNA was isolated from dASCs using RNeasy Plus Mini Kit (Qiagen, Manchester, UK), according to the manufacturer’s protocol. Each sample was reverse-trascripted using RT^2^ First Strand Kit (Qiagen, Manchester, UK), according to the manufacturer’s protocol. cDNA was used in RT-PCR and primers and GoTaq Green Master Mix (Promega, Madison, WI, USA) were added. For semiquantitative RT-PCR the densitometric analysis of the bands were performed using ImageJ software (NIH, Bethesda, MA, USA) (OD amplicon/OD housekeeping gene). These values are expressed as arbitrary units. Quantitative real-time PCR was performed with RT^2^ SYBR Green qPCR Mastermix (Qiagen, Manchester, UK) using Corbett Rotor Gene 6000 real-time cycler (Qiagen, UK). All reactions were carried out in triplicate and the protocol used was: hot start for 10 min at 95 °C, followed by 45 cycles of 15 s at 95 °C, annealing for 30 s at 55 °C and extension for 30 s at 72 °C. The sequences of the primers used are reported in Table 1. Data were normalised with housekeeping gene (18S or gapdh) and the ΔΔCt method was used to determine the fold changes in the gene expression, as compared to control.

**Table 1:** Primer sequences used in semiquantitative and quantitative RT-PCR analysis

Gene	Forward	Reverse
*mAChR M1*	5′-CCATGGAGTCCCTCACATCCT-3′	5′-ATCTACCATGGGCATCTTGATCA-3’
*mAChR M2*	5′-GCTCCAATGATTCGACGTCA-3′	5′-CGAAGTGGAAACTGTTGTTTTCAT-3′
*mAChR M3*	5′-TCCATCCTCAACTCTACCAAGCT-3′	5′-TTGTGAGCATTTCTCTCCACATC-3′
*mAChR M4*	5′-CACTCTGCAATGCCACTTTCAA-3′	5′-CTGTGCCGATGTTCCGATACT-3′
*mAChR M5*	5′-ACCCGCACTGAAAACAGTGACT-3′	5′-ATCGGAACTAGGCAACACACTT-3′
*CD29*	5′-TGGGTCGCTGATTGGCTG-3′	5′-CTCTTCAGTGACTGCAAAAATCG-3′
*CD44*	5′-TCATGTTAGAGCATCCGTGC-3′	5′-GGGTTGTACATCATGCCTCC-3′
*CD90*	5′-TGAACCCAGTCATCAGCAT-3′	5′-CAGTCGAAGGTTCTGGTTCACC-3′
*cyclinD1*	5′-CCCTCTGCACGCACTTGAAG-3′	5′-GCGAGCCATGCTTAAGACTGA-3′
*pcna*	5′-GAAGCACCAAATCAAGAGAA-3′	5′-TCACCCCGTCCTTTGCACAG-3′
*notch1*	5′-CTCACGCTGATGTCAATGCT-3′	5′-GCAACACTTTGGCAGTCTTCA-3′
*c-jun*	5′-GATGGAAACGACCTTCTACGAC-3′	5′-AGCGTATTCTGGCTATGCAGTT-3′
*sox10*	5′-ACTGGGAACAGCCAGTATATA-3'	5′-ACCAAACTCCTCCTTTGCCA-3'
*s100B*	5′-ATAGCACCTCCGTTGGACAG-3'	5′-TCGTTTGCACAGAGGACAAG-3'
*gfap*	5′-GCTGAACTGAACCAGCTTCGA-3'	5′-CTTGGCCACATCCATCTCCAC-3'
*p0*	5′-TCTTTTACCTGGCGCTACCAG-3'	5′-GTTGACCCTTGGCATAGTGGA-3'
*egr2*	5′-AACGGAGTGGCCGGAGAT-3′	5′-ATGGGAGATCCAACGACCTCTT-3′
*nrgI/I*	5′-TCATCTTCGGCGAGATGTCTG-3'	5-‘CTCCTGGCTTTCATTTCTTTCA-3'
*nrgI/III*	5′-GGACCCCTGAGGTGAGAGAACA-3'	5′-CAGTCGTGGATGTCGATGTGG-3'
*erbB2*	5′-CGAGTGTCAGCCTCAAAACA-3'	5′-CTCATCCGGGTACTTCCAGA-3'
*erbB3*	5′-CTGTTTAGGCCAAGCAGAGG-3'	5′-GACTTTGTTTGCCTTCTCGC-3'
*CXCR4*	5′-GCCATGGCTGACTGGTACTT-3′	5′-GATGAAGGCCAGGATGAGAA-3′
*CXCR7*	5′-GGCTACGACACACACTGCTA-3′	5′-GGTCCACGCTCATGCATGCG-3′
*CXCL12*	5′-TGCATCAGTGACGGTAAGCCA-3′	5′-ATCCACTTTAATTTCGGGTCAA-3′
*18* *S*	*5*′*-GGATCCATTGGAGGGCAAGT-3*′	*5*′*-ACGAGCTTTTTAACTGCAGCAA-3*′
*Gapdh*	*5*′*-GTGCCAGCCTCGTCTCATAG-3*′	*5*′*-TGATGGCAACAATGTCCACT-3*′

### Western blot

Whole-cells lysates were obtained by scraping cells from confluent 6 well-plates using RIPA Buffer (Sigma-Aldrich, UK) supplemented with a cocktail of protease and phophatase inhibitors (Thermo Scientific, Loughborough, UK). Lysates were incubated for 30 min on ice and then centrifuged for 20 min at 14 rpm at 4 °C. Protein concentration was determined using Pierce™ BCA Protein Assay Kit (Thermo Scientific, Waltham, MA, USA), according to the manufacturer’s protocol. Sample buffer (6 × ) was added to protein lysates and heated for 5 min at 100 °C. Afterwards, 30 µg of the sample was loaded onto a 10% SDS (Sodium dodecyl sulfate) polyacrylamide gel and run at 120 V using running buffer (25 mM Tris, 190 mM glycine, 0,08% (w/v) SDS). SDS-PAGE gels were transferred for 1 h onto nitrocellulose blotting membranes (GE Healthcare Life Science, Amersham, Germany) at 80 V in transfer buffer (25 mM Tris-base; 192 nM glycine, 20% (v/v) methanol). After transfer, membranes were blocked for 1 h in a Tris-buffer saline (TBS)- Tween Solution containing 5% non-fat dry milk. Membranes were incubated with the primary antibody, diluted in blocking buffer, overnight at 4 °C. Primary antibodies used were: Mouse anti-M2 antibody (1:500, Abcam, UK), rabbit anti-c-Jun (1:1000, Abcam, UK), rabbit anti-Egr-2 (1:500, ProteinTech, UK). After overnight incubation, membranes were washed with TBS-Tween buffer and thus incubated for 1 h at room temperature with secondary antibody: anti-rabbit horseradish peroxidase (1:2000, Cell Signaling, Hitchin, UK) or anti-mouse horseradish peroxidase (1:1000, Cell Signaling, Hitchin, UK) for chemiluminescence detection. Actin or β-tubulin was used as protein reference (mouse anti-actin, 1:6000, (Millipore), rabbit anti-β-tubulin (1:1000, Abcam, Cambridge, UK)). To determine housekeeping protein, membranes were stripped before re-blotting with another primary antibody. Membranes were exposed to SuperSignal West Pico Chemiluminescent Substrate (Thermo Scientific, Waltham, MA, USA) for signal detection.

### Immunocytochemistry

Cells were seeded into 35 mm^2^ plates in complete medium at the density of 2 × 10^4^/ml.

At subconfluence, cells were washed twice with PBS and fixed with 4% PFA (paraformaldehyde, PFA) for 20 min at room temperature (RT). Cells were incubated with Triton-X 0,2% for 30 min at RT. Then cells were washed twice with PBS and treated with block solution (PBS 0.1% Triton X-100 and 10% normal donkey serum (NGS) for 1 h at RT. The cells were incubated with anti-rabbit-GFAP (1:100, DAKO, Glostrup, Denmark) and anti-rabbit-S100β (1:100, DAKO, Glostrup, Denmark) at 4 °C overnight. The day after, cells were washed with PBS three times for 10 min and they were incubated with goat anti-rabbit IgG Alexa Flour 488-conjugated antibody (Life Technologies, UK), diluted 1:500 in 0.1% Triton X-100, 0,1% (w/v) BSA, 0,1% (w/v) Sodium Azide in PBS for 1 h at RT. After, plates were washed 3 times with PBS and slides were mounted with Vectashield mounting medium for fluorescence containing 4’-6’-diamidino-2-phenylindole for nuclear staining (H1200, Vector Lab, DBA, Milan, Italy). Images were taken using a fluorescence microscope (Olympus IX51, Sounthend-on-Sea, UK) and processed with ImageJ 64 imaging software (National Institutes of Health, NIH, Bethesda, MD, USA).

### Measurement of cAMP levels

Cells were seeded on 6-well plate at the density of 25 × 10^3^ cells/well. When cells were at 80% confluent, they were treated with APE 100 μM. Each condition was done in triplicate and samples from the same groups were pooled. cAMP analyses were performed using cyclic AMP Select ELISA kit (Cayman Chemical, MI, USA), according to the manufacturer’s protocol. Plates were read with an Asys UVM-340 microplate reader/spectrophotometer (Biochrom, Cambridge, UK). Absorbance was recorded at 420 nm.

### Proliferation assay

To study cell proliferation, dASCs were seeded on 24-well plates at the density of 25 × 10^3^ cells/well. After 24 h, cells were treated with APE 100 μM (Sigma-Aldrich, St. Louis, MO, USA) for 3, 5, 7 days. M2 muscarinic receptor antagonist methoctramine (10^−7^ M, Sigma-Aldrich, St. Louis, MO, USA) was used 2 h before APE treatment, when required. This method is based on a tetrazolium compound reduction generating a colored formazan product by viable cells; this is soluble in cell culture media. Cells were incubated in 20% (v/v) CellTiter 96 AQueous One Solution Cell Proliferation Assay (Promega, Southampton, UK), diluited in phenol-free DMEM (Sigma-Aldrich, UK) for 90 min at 37 °C in the dark. For each well, the absorbance at 490 nm was recorded with an Asys UVM-340 microplate reader/spectrophotometer (Biochrom, Cambridge, UK). After a standard curve was performed, data were expressed as cell number ± SEM of the mean.

### Live dead assay

Cell viability was analysed by using LIVE/DEAD Viability/Cytotoxicity Kit for mammalian cells (Molecular Probes, Invitrogen, UK). Calcein-AM was used to identify live cells (green fluorescence; λ = 498 nm), conversely Eth-D1 was used to identify dead cells (red fluorescence; λ = 635 nm). dASCs were plated in a concentration of 40 × 10^3^ cells/well into 12 well plates. Calcein-AM and Eth-D1 were added at a concentration of 0.5 μl/ml and 2 μl/ml, respectively, and cells were incubated for 30 min at RT, and then washed in PBS prior to imaging. Images were taken using fluorescence inverted microscope (Olympus IX51, Sounthend-on-Sea, UK) and processed with ImageJ64 imaging software (NIH, Bethesda, MD, USA) under 4x magnification.

### Recovery assay

Recovery analysis was set up to demonstrate whether inhibition of proliferation-APE induced was reversible. The cells were treated for 72 h with APE. Then, complete medium containing APE was removed; the cells were washed once with sterile PBS, and fresh complete media added. Growth analysis was assessed as described above. For each well, the absorbance at 490 nm was recorded with an Asys UVM-340 microplate reader/spectrophotometer (Biochrom, Cambridge, UK). Stardard curve was performed and the data were expressed as cell number ± SEM of the mean.

### Wound healing

Cell migration was evaluated into six-well plates in triplicate for all conditions. Wound was generated with p200 tip and, to exclude possible proliferation interference, Mitomycin C was also added to the cell culture media (50 ng/ml, Sigma-Aldrich). The cells were photographed at time 0 and after 6 h at 4x magnification. The space was measured between two fronts at time 0 h (T0) and 6 h (T6). The two values were subtracted, obtaining the covered space by the cells in the experimental time chosen. After 6 h, cells were fixed with 4% PFA in PBS (Sigma-Aldrich, UK), permealized for 15 min with 0.2% (v/v) Triton-X in PBS (Sigma-Aldrich, UK) and stained for 20 min at RT with Alexa 488-conjugated phalloidin (1:40, Life Technologies, UK) diluted in 1% (w/v) BSA in PBS. Images were taken using a fluorescence microscope at 4x magnification (Olympus IX51, Sounthend-on-Sea, UK) and processed with ImageJ 64 imaging software (NIH, Bethesda, MD, USA).

### Cell morphology assessment

To determine individual cell morphology dASCs were plated in six-well plates in triplicate at a density of 50 × 10^3^ cells/well. After 24 h, cells were treated with APE 100 μM (Sigma-Aldrich, St. Louis, MO, USA) or APE plus M2 antagonist methoctramine (Meth10^−7^ M, Sigma-Aldrich, St. Louis, MO, USA). The time points chosen were from 1 to 3 days after treatment.

Images were captured with an Olympus IX51 wide-field microscope at 10x magnification. Images were analysed with IMAGE J (v1.47f; NIH, Bethesda, MD, USA) to measure cell length (longest cell length) and width (narrowest cell width). The *aspect ratio* was calculated as length/width for each cell measured.

### Statistical analysis

Data analysis were performed with GraphPad Prism (ver 7.0, GraphPad Software Inc, La Jolla, CA, USA). Data were presented as the mean ± SEM. Student’s *t*-test or one-way ANOVA analyses with Bonferroni post-tests were used. A value of *p* < 0.05 was considered statistically significant. (*p* < 0.05 (*), *p* < 0.01 (**) and *p* < 0.001 (***)).
